# Health-Related Quality of Life (HRQoL) of Patients with Tuberculosis: A Review

**DOI:** 10.3390/idr14040055

**Published:** 2022-07-18

**Authors:** Sandul Yasobant, Mahalaqua Nazli Khatib, Zahiruddin Quazi Syed, Abhay M. Gaidhane, Harsh Shah, Kiran Narkhede, Priya Bhavsar, Jay Patel, Anish Sinha, Tapasvi Puwar, Somen Saha, Deepak Saxena

**Affiliations:** 1 Department of Public Health Science, Indian Institute of Public Health Gandhinagar (IIPHG), Gandhinagar 382042, India; yasobant@iiphg.org (S.Y.); hdshah@iiphg.org (H.S.); drkirannarkhede@gmail.com (K.N.); priyabhavsar@iiphg.org (P.B.); jpatel@iiphg.org (J.P.); asinha@iiphg.org (A.S.); tpuwar@iiphg.org (T.P.); ssaha@iiphg.org (S.S.); 2 School of Epidemiology and Public Health, Datta Meghe Institute of Medical Sciences (Deemed to be University), Wardha 442004, India; nazli.786@rediffmail.com (M.N.K.); zahirquazi@gmail.com (Z.Q.S.); abhaygaidhane@gmail.com (A.M.G.)

**Keywords:** HRQoL, QoL, tools, assessment, factors, TB

## Abstract

Tuberculosis (TB) is a major killer and cause of human suffering worldwide and imposes a substantial reduction in patients’ health-related quality of life (HRQoL). HRQoL indicates the consciousness of patients regarding their physical and mental health. It is, therefore, very relevant in comprehending and measuring the exact impact of the disease state. Therefore, we undertook this review to summarize the available evidence on the impact of TB and its treatment on HRQoL. An in-depth understanding of HRQoL in TB patients can identify the existing management gaps. We undertook a systematic search through PubMed and CENTRAL. Data were extracted and tabulated for study design, targeted population, QoL instrument used, QoL domain assessed, and key findings. We included studies that assessed the effect of TB on the QoL both during and after treatment. There are no specific HRQoL assessment tools for utilization among TB patients. HRQoL is markedly impaired in patients with TB. The factors affecting HRQoL differ with active and latent TB, socio-demographics, socio-economic status, presence of co-infections, etc. This review’s findings can help to frame appropriate policies for tackling HRQoL issues in TB patients.

## 1. Introduction

Tuberculosis (TB) is the number one infectious killer worldwide, ranking above HIV/AIDS. TB is a significant concern for public health as a key reason for morbidity and mortality, resulting in nearly 4000 deaths a day. Regardless of advancements in identification and management, approximately 10 million people developed TB, and an estimated 1.4 million people died from TB globally in 2019 [1,2]. About one-fourth of the global population has been infected at least once with Mycobacterium tuberculosis in their lifetime [3]. Higher risk is reported in people living with HIV and comorbidities such as malnutrition, diabetes mellitus, substance abuse, etc. TB patients may exhibit a broad range of symptoms, from asymptomatic to symptoms involving single-organ or total manifestations. Fever and cough are the most common reported symptoms [4]. Patients are known to suffer due to the disease symptoms and subsequent decline in patient health-related quality of life (HRQoL) [5,6]. The disease adversely affects all quality of life domains, ranging from physical, economic, and social to psychological distress resulting from stigmatization and discrimination associated with the disease [7]. Quality of life (QoL) is a comprehensive and multifaceted idea that integrates physical, social, mental, economic, and other domains. It defines the satisfaction sensed by an individual and measures the patient’s self-perceived health status, functioning, and overall well-being [8]. As QoL emphasizes an “individual’s perception of their position in life in the context of the value systems and culture in which they live and in relation to their expectations, goals, standards, and concerns”, it becomes difficult to assess [9]. Therefore, rather than assessment by healthcare providers, QoL should reflect the inclination and values of the patient. As HRQoL indicates the consciousness of patients regarding their mental and physical health, it is very relevant in comprehending and measuring the exact impact of the disease. However, the existing TB identification and management approaches depend on clinical courses and microbiology, and there is no room to evaluate patients’ physical and psychological health.

With scientific advances in the management strategies for TB, an increasing number of patients can survive the disease. Thus, a change in TB patients’ routine assessment of clinical status and microbiological outcomes is essential. Efficient management of disease relapse, in addition to the appearance of multidrug-resistant tuberculosis (MDR-TB), is linked to the adherence to anti-TB treatment (ATT) and subsequently to HRQoL [10]. The assessment of patient-reported outcomes (PROs) provides evidence about the experience of living with the diseased condition and goes beyond clinical parameters, encompassing physical, mental, and social well-being. Thus, nowadays, PROs are better acknowledged and appreciated in managing diseases and evaluating disease outcomes [11]. Evaluation of HRQoL using PROs permits a complex interpretation of health and aids in measuring the efficacy of treatment on a patient’s health and daily life.

There is a dearth of evidence on assessing HRQoL among TB patients. Further, the effect of a load of TB and ATT on HRQoL in TB patients has been underattended. Systematic reviews by Bauer et al., Chang et al., and Guo et al., [6,7,12] were undertaken several years back. Therefore, we undertook this review to summarize the available evidence on the impact of TB and its treatment on HRQoL. An in-depth understanding of HRQoL in TB patients can identify the existing management gaps. Bridging these gaps will improve and enhance healthcare services and provide provisions for framing health policies. The findings generated in this review can help a range of stakeholders, including patients, caregivers, healthcare providers, practitioners, patient support groups, policymakers, and non-governmental organizations, in designing new approaches and addressing gaps in the care cascade. 

## 2. Methodology

We undertook a systematic search through PubMed and CENTRAL in November 2020 using keywords such as “tuberculosis” and “quality of life” (((tuberculosis [Title/Abstract]) OR (TB [Title/Abstract])) OR (koch*[Title/Abstract])) OR (“tuberculosis” [MeSH Terms]) AND ((“quality of life” [Title/Abstract]) OR (QoL [Title/Abstract])) OR (“quality of life” [MeSH Terms]). We additionally searched for key references from bibliographies of the relevant studies. The search was updated in February 2022.

**Figure 1:**
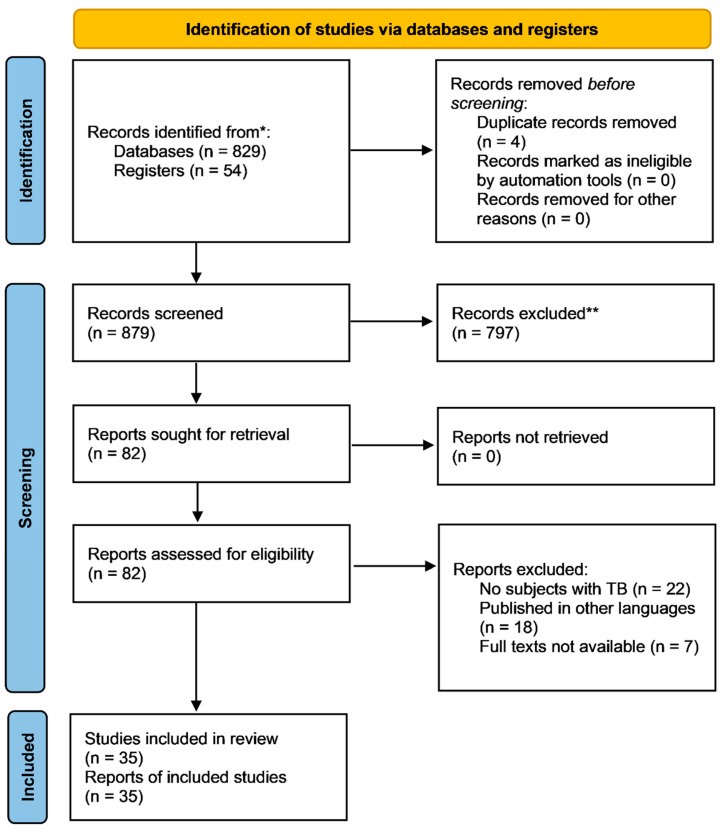
PRISMA Flow Diagram for QoL and TB. * Consider, if feasible to do so, reporting the number of records identified from each database or register searched (rather than the total number across all databases/registers); ** If ambulation tools were used, indicate how many records were excluded by a human and how many were excluded by automation tools.

One reviewer independently monitored the retrieved studies against the inclusion criteria, in the beginning, based on title and abstract and then on full texts. Another reviewer also reviewed approximately 20% of these studies to validate the inclusion of studies. Differences were resolved through discussion. One reviewer extracted the data from studies according to the mental, physical, and social health aspects of HRQoL. Data were extracted and tabulated on details about the study design, population, QoL instrument used, QoL domain assessed, and study findings. We included studies that assessed the effect of TB on QoL both during and after treatment irrespective of the study design, geographic location, age, gender, type of TB, or type of treatment. For inclusion, both published and unpublished studies in the English language were considered. We excluded studies that were published in other languages because of resource limitations or if the full-text articles were unavailable to reviewers. We also excluded studies other than TB or TB has been considered as one of the comorbidity along with any other chronic conditions or HR-QoL was not assessed, as shown in Figure 1. 

## 3. Findings

We retrieved 829 initial hits from the literature search in PubMed and CENTRAL. Additional electronic searches, a bibliography of relevant studies, and hand searches yielded 54 studies. Most of the research has been focused on clinical outcomes; only a few studies have scrutinized the impact of tuberculosis on patient quality of life.

### 3.1. Instruments for Describing and Quantifying QoL in TB

Different questionnaires and scales have been used to assess self-rated HRQoL in TB patients and patients infected with nontuberculous mycobacteria (NTM) [6,7,13,14]. Some questionnaires assess HRQoL holistically, while others assess specific domains, such as physical or emotional domains. HRQoL instruments can be generic or disease-specific. Standardized instruments to assess HRQoL have been used in TB patients [15,16,17]. Frequently used tools to assess QoL in TB patients include the Short Form 36 (SF-36) [5,11,15,16,17,18,19,20,21,22,23,24,25,26,27,28,29,30,31], the EQ-5D, [16,24,32,33,34,35], and the abbreviated World Health Organization Quality of Life scale (WHOQOL-BREF) [36,37,38,39,40,41,42]. TB-specific QoL tools have not been extensively used.

### 3.2. Factors Associated with HRQoL in TB

Several studies have found that overall well-being, including HRQoL, is strikingly compromised in patients with TB as compared to healthy people across many domains [7,12,15,17,19,20,24,30,33,36,37,40,43,44,45,46,47]. Both general and precise QoL tools showed a broad spectrum of imbalances in scores and outcomes that differed across different countries and different patient groups. The included studies assessed factors associated with HRQoL in TB patients such as socio-demographic factors (age, gender), socio-economic factors (income, education, housing, social security), presence of co-infections (especially HIV) or comorbidities (such as diabetes, anemia), factors associated with ATT (adverse drug reactions), and psycho-social aspects (isolation and stigmatization) [6,7,11,15,21,22,34,36,37,40,44,46,48,49,50,51,52,53,54,55]. Few studies found a larger impact of psycho-social burden than clinical symptoms in TB patients [12,44]. 

#### 3.2.1. Active and Latent Tuberculosis (LTBI)

Several studies have found that patients with active TB reported poorer HRQoL as compared to patients with LTBI or previously cured TB or untreated individuals or healthy controls [5,6,11,16,21,24,31,40,44,56,57,58,59,60] (Table 1). The psychological domain was more negatively affected than the physical domain among patients with active and latent TB [6]. The HRQoL scores of LTBI patients were normal and similar to those in the general population [59]. The number of people reporting problems was lower in LTBI patients than in patients with active TB [61]. A meta-analysis by Bauer et al. [7] depicted a major improvement in HRQoL during the initial treatment period in patients with active TB.

#### 3.2.2. Socio-Demographic (Age, Gender, and Others) and Socio-Economic (Income, Education, Housing, Social Security) Status

Overall, QoL seems largely independent of age and gender [62,63]. However, some studies reported that advancing age negatively correlated with QoL [21,34,36,48]. Among women, others reported worse QoL [30,36,40]. One study reported that males have unfavorable outcomes in the psychological domain of HRQoL [48]. A study conducted on the Indian subcontinent observed high HRQoL scores in females for the physical and environmental domains, perhaps indicative of improved managing strength in females [40]. Similarly, illiteracy and a low socio-economic state can be linked with larger derangements of HRQoL [31,62]. Higher education had a significant positive impact on the mental domain of HRQoL [64]. The HRQoL differed between developing and developed countries concerning their socio-demographic and economic conditions [65,66]. Tuberculosis had a socio-economic impact on patients and their families. One study found that female TB patients had compromised caregiving abilities, leading to one-fifth of school-going children discontinuing their studies [67].

#### 3.2.3. QoL in Tuberculosis with HIV Co-Infection

In high-burden settings, HIV co-infection is one of the key risk factors for tuberculosis development, escalating the susceptibility to primary infection, reinfection, and risk of reactivation of TB in patients with latent TB. Thus, we assessed QoL in TB with HIV co-infection. Several studies included patients co-infected with TB and HIV [34,52,54,68,69,70,71,72,73,74] (Table 2). Studies [70,71,75] reported lower QoL (physical and mental scores) in people co-infected with HIV and TB compared to TB patients. Few studies [72] found similar scores in HRQoL domains between individuals that received antiretroviral therapy (ART) with ATT and those that received ATT only [69,72].

#### 3.2.4. QoL in Tuberculosis with Other Comorbidities 

Studies have found that comorbidities such as undernutrition or diabetes mellitus (DM) impact the TB care cascade [76,77]. For other diseased conditions, comorbidities or concomitant treatment is prognostic of lower QoL scores, especially in the physical domain [48]. There is insufficient evidence that the presence of comorbid conditions additionally worsens HRQoL (Table 3).

### 3.3. QoL during and after Treatment 

The WHO reports that the annual number of TB patients accessing ATT increased from 6 million in 2015 to 7.1 million in 2019 [3]. Several studies have demonstrated that ATT resulted in substantial improvement in HRQoL [4,5,6,7,11,15,22,24,36,45,50,51,54,55,78,79,80] (Table 4). Although HRQoL improves during ATT, most TB patients continue to demonstrate some impairment in HRQoL. While all domains of HRQoL were compromised in TB patients, the maximum impact was observed on the mental domain and least on the physical domain, particularly in the early months of treatment [5]. Integrated TB treatment strategies, such as the TB-tobacco treatment strategy, have been shown to potentially improve overall QoL outcomes among TB patients [50]. HRQoL scores differ with the type of treatment and may be used to identify possible defaulters during ATT [8]. A systematic review by Kastien-Hilka et al. found that, although ATT improved all HRQoL domains, the psycho-social domain remained impaired after treatment [80]. Results of a systematic review [80] suggest that patient-reported HRQoL outcomes may vary after the completion of treatment depending on the HRQoL measures. Studies have indicated that, although HRQoL improved with treatment, it was compromised in TB patients at the initiation and completion of treatment [5,19,24]. The best improvements in HRQoL were observed during the intensive phase of ATT [7]. One study suggested that the HRQoL was low at the completion of ATT [11]. Some studies suggested significant improvements in HRQoL after six months of ATT [29,81]. After the successful completion of ATT, HRQoL scores of TB patients were almost at the same levels as those of the general population [29,82]. Adverse drug reactions (ADR) from ATT may sometimes deteriorate HRQoL more in the mental dimension than the physical dimension [22,28,83,84]. Gastrointestinal disturbances, visual impairment, or peripheral neuropathy associated with ATT may negatively affect HRQoL [44]. Patients with low pre-treatment HRQoL scores demonstrated higher adverse effects than those with normal pre-treatment HRQoL [22]. 

Studies on HRQoL in MDR-TB and extensively drug-resistant tuberculosis (XDR-TB) patients are very limited. Despite the positive impact of MDR-TB treatment on HRQoL, physical health, mental health, and social functioning are compromised in MDR-TB patients, even after the completion of ATT, as compared to drug-susceptible tuberculosis [18,28,84,85]. Patients with relapse or re-treatment are likely to demonstrate more derangements in QoL [40].

**Table 4:** Characteristics of studies that assessed QoL in TB during and after treatment.

Study ID (Country)	Study Design	Participants	QoL Instrument Used	QoL Domain Assessed	Findings
Kastien-Hilka et al., 2016 (South Africa) [80]	Systematic review	TB patients	1. WHOQOL-BREF2. SGRQ 3. SF-36 4. EQ-5D E 5. BDI 6. STAI-6 7. CES-D	1. Physical 2. Mental 3. Social health	Although ATT improved all HRQoL domains, the psycho-social domain remained impaired after treatment
Bauer et al., 2015 (Canada) [5]	Longitudinal cohort study	1. TB 2. LTBI 3. Control participants	SF-36	1. Physical component summary 2. Mental component summary	TB patients have lower HRQoL scores; Higher decrements in HRQoL are observed soon after starting ATT but improve after two months of ATT
Atif et al., 2014 (Malaysia) [19]	Longitudinal study	New smear-positive PTB patients	SF-36	1. Physical 2. Mental health	Compromised mental and physical health was observed among TB patients even after the completion of treatment
Chung and Li, 2013 [79]	Prospective cohort study	TB patients undergoing treatment	WHOQOL-BREF	1. Physical 2. Mental 3. Social 4. Environmental	TB affects HRQoL despite effective treatment
Aggarwal et al., 2013 (India) [36]	Cohort study	Newly diagnosed PTB patients starting treatment (RNTCP)	WHOQOL-BREF	1. Physical 2. Psychological 3. Social relationships 4. Environment	For patients treated by the RNTCP, HRQoL assessment may be used as an adjunct outcome measure
Ralph et al., 2013 (Indonesia) [55]	Clinical trial	1. Smear-positive TB 2. Healthy control	SGRQ Indonesian version	1. Symptoms component 2. Impact components (social functioning, psychological disturbances resulting from airways disease)	PTB treatment can reduce symptoms, improve functional capacity, and improve QoL
Balgude and Sontakke, 2012 [51]	NA	1. Newly diagnosed pulmonary TB 2. Healthy control	WHOQOL-BREF	1. Physical health 2. Psychological health 3. Social relationships 4. Environment	Even after ATT, physical and psychological domains remain lower in TB patients than in controls
Awaisu et al., 2012 (Malaysia) [50]	Multi-centered non-randomized controlled study	1. TB-DOTS plus SCI 2. TB-DOTS only	1. EQ-5D 2. EQ-VAS	1. Mobility 2. Self-care, 3. Pain or discomfort 4. Anxiety or depression	An integrated TB-tobacco treatment strategy can improve HRQoL among smoker TB patients
Guo et al., 2010 (Canada) [22]	Longitudinal study	1. Active TB patients 2. LTBI patients	SF-36	1. PCS 2. Mental component summary (MCS)	Improvements in the baseline HRQoL of high-risk patients can support the outcome of medical treatment
Kruijshaar et al., 2010 (UK) [24]	NA	TB patients	SF-36 v2UK versionEQ-5DSTAI-6 CES-D	1. Physical and general health 2. Vitality and mental health. 3. Anxiety and depression	ATT improved HRQoL but were lower than normal people.
Guo et al., 2009 [6]	Systematic review	TB patients	1. SF-36 2. GQOLI-74 3. QLQ 4. SGRQ 5. SSRS 6. BDQ	1. PCS 2. MCS	TB has impact on patients’ QoL even after treatment
Marra et al., 2008 [11]	NA	1. Active TB patients 2. LTBI patients	SF-36 v2BDI	1. Physical 2. Mental 3. Social 4. Emotional	Active TB patients showed improvements in most HRQoL domains
Muniyandi et al., 2007	NA	(TB) patients one year after treatment completion.	SF-36	1. Physical 2. Mental 3. Social well-being	HRQoL of TB patients after one year of completion of ATT was normal
Pasipanodya et al., 2007b (United States) [86]	NA	PTB or latent tuberculosis infection (LTBI)	SGRQ	1. Symptoms component 2. Impact components (social functioning, psychological disturbances resulting from airways disease)	PTB has a substantial impact on human health, thus affecting QoL
Dion et al., 2004 (Canada) [16]	Cross-sectional study	Pulmonary tuberculosis (PTB)	SF-36v2 SF- 12v2	1. PCS 2. MCS	Most cured TB patients have compromised HRQoL

ATT: anti-tuberculosis treatment; HRQoL: health-related quality of life; PTB: pulmonary tuberculosis; RNTCP: Revised National TB Control Program; MCS: mental component summary; PCS: physical component summary.

## 4. Discussion

In this review, we collated data from multiple primary studies to furnish fresh insights into HRQoL in TB patients that can be useful to healthcare providers, policymakers, and patient support groups in designing newer approaches and addressing gaps in patient care.

Due to the lack of validated TB-specific instruments for assessing HRQoL, a wide range of tools have been used in TB patients [13]. Studies suggest that overall well-being and HRQoL are clearly impaired across all domains in patients with TB [12,17,36,44,46]. Economic burden due to reduced work capacity, social stigmatization, and psychological issues may deteriorate HRQoL in this population [68]. Several studies have found that subjects with active TB had poorer HRQoL than patients treated for LTBI and untreated individuals [5,6,7,11,21,24,31,40,44,57,58,59]. In contrast to active TB, LTBI is usually asymptomatic and does not affect HRQoL. However, diagnosis and treatment for LTBI can be linked with adverse events and mental health challenges. Studies have reported lower HRQoL in co-infected (TB-HIV) individuals as compared to patients with TB [34,54,68,70,71,73,75]. TB co-infection worsens the symptoms of HIV and advances the disease to the next stage, which can further deteriorate the HRQoL [70]. Additionally, studies have found that TB patients are negatively impacted by comorbidities such as undernutrition, diabetes mellitus (DM), anemia, or HIV infection [76,77]. 

This measure is important in evaluating and measuring the actual effect of disease on patients. Poor baseline HRQoL status may indicate physical and mental health issues, which can lead to a greater risk of undesirable outcomes. Thus, self-reported HRQoL can be considered as an adjunct to disease outcomes and not merely an alternative tool. Lower HRQoL scores have been reported among TB patients at the start of treatment as well as after the completion of ATT [5]. Though all of the HRQoL domains were affected, maximum derangements were observed in mental health dimensions and lowest derangements in physical health dimensions [5]. Although ATT improved HRQoL scores, they remained below normal [5,19,24,87]. HRQoL scores differ with the course of treatment and may even help to recognize possible defaulters during treatment [8]. The possibility of experiencing adverse drug reactions is higher in TB patients with low baseline (pre-treatment) HRQoL scores than those with normal baseline (pre-treatment) HRQoL scores [22]. This implies that HRQoL assessments at baseline (pre-treatment) can help the healthcare provider in recognizing patients likely to develop an ADR to ATT.

TB is known to be a significant cause of mortality and morbidity across the globe. However, the detrimental effects of TB and its treatment on HRQoL are immense and have been unattended. There is scope for considering the assessment of HRQoL at diagnosis as an adjunct outcome measure for evaluation and outcome measure for patients under ATT [11]. The evaluation of the link between HRQoL and adherence to ATT may provide important evidence on treatment efficiency, optimal management of TB patients, and policymaking. These findings call the attention of healthcare providers to tackle issues related to HRQoL in TB patients. The evidence generated in this review emphasizes a possible role in identifying any impairments in HRQoL at diagnosis and providing timely holistic care and patient-centered approaches for improving HRQoL in TB patients.

The authors recommend that concerns related to compromised HRQoL should be dealt with at diagnosis and during treatment in TB patients. Assessment of HRQoL may need to be introduced as an adjunct outcome measure into treatment protocols for TB. Monitoring of HRQoL during follow-up treatment of TB may be necessitated. Patients who may require monitoring can be identified by obtaining HRQoL scores and distinguishing features of defaulters. This can help in tackling the issue of defaulters in TB treatment. Integration of strategies (such as mental health support, socio-cultural reforms, and health education) to improve HRQoL issues is strongly needed to optimize TB care and treatment outcomes.

### 4.1. Study Limitations

Most studies included in this review were cross-sectional and undertaken in developing countries with the greatest burden of TB. Most studies included participants from different socio-economic strata and did not provide socio-demographic details. Explicit descriptions of these details of the participants in the included studies would have made it possible to extract similarities and differences between studies. This would have provided better insights into generalizability and transferability to other settings. The protocol was not registered anywhere. 

### 4.2. Implications for Policy, Practice, and Research

The evidence generated in this review emphasizes the importance of assessing HRQoL in TB patients and encourages decision-making regarding treatment and the possible role of providing timely holistic care approaches in improving HRQoL tailored to patient needs. This review’s findings can help to frame appropriate policies for tackling HRQoL issues in TB patients.

The findings generated in this review point to further research to understand the effect of TB on HRQoL and to develop holistic care approaches to improve the same. Prospective studies can be undertaken to understand the prognostic value of HRQoL further and to study whether this assessment can be used to manage TB patients. Further research on HRQoL in the context of TB is needed to address social and behavioral determinants. Research could also be carried out on vulnerable populations, such as those with TB/HIV co-infection or MDR-TB, young populations, and pregnant women.

## 5. Conclusions

There are no precise HRQoL assessment tools for TB patients. The authors recommend that a TB-specific QoL assessment tool be developed, standardized, and validated. Poorer HRQoL had been reported in patients with active TB compared to patients with latent TB or previously cured TB, untreated individuals, or healthy controls. Patients co-infected with TB and HIV reported lower QoL as compared to TB patients. There is insufficient evidence as to whether the presence of comorbid conditions additionally worsens HRQoL. Anti-TB treatment can lead to improvement in HRQoL. However, adverse drug reactions from ATT may sometimes provoke the deterioration of HRQoL. This review’s findings can help to frame appropriate policies for tackling HRQoL issues in TB patients.

## Figures and Tables

**Table 1:** Characteristics of studies that assessed QoL in active and latent tuberculosis (LTBI).

Study ID (Country)	Study Design	Participants	QoL Instrument Used	QoL Domain Assessed	Findings
Shedrawy et al., 2019 (Sweden) [59]	Cross-sectional study	LTBI	1. EQ-5D-3 L 2. RHS-15	1. Physical, 2. Psycho-social 3. Pain	No HRQoL decrements were detected in LTBI patients
Bauer et al., 2015 (Canada) [5]	Longitudinal cohort study	1. Diagnosed and treated for TB disease 2. LTBI 3. Persons screened but not treated for TB disease or LTBI	SF-36	Eight domain scores and mental and physical component summary	HRQoL decrements detected in TB patients
Bauer et al., 2013 [7]	Systematic review and meta-analysis	1. Active TB 2. LTBI 3. Healthy controls treatment	1. SF-36 2. GHQ–12 3. HAD 4. MACL 5. SIP 6. SRI (BDI) 7. EQ 5D 8. WHOQOL-BREF 9. WHOQOL-HIV	1. Physical 2. Psychological 3. Social 4. Environment	Poorer HRQoL was reported in subjects with active TB than in persons treated for LTBI
Kruijshaar et al., 2010 (UK) [24]	Prospective study	Pulmonary TB patients	1. SF-36 2. EQ-5D	1. Physical 2. Psychological	TB patients had diminished HRQoL scores
Guo et al., 2009 [6]	Systematic review and meta-analysis	1. Active TB 2. LTBI, regardless of the treatment status	1. SF 36 2. HUI, 3. EQ-5D 4. VAS 5. QLQ 6. GHQ-12 7. BDQ 8. SSRS	1. Physical 2. Mental 3. Social well-being 4. Functioning	1. HRQoL decrements in TB patients 2. Among patients in both active and LTBI, mental well-being was more severely disrupted than physical health
Marra et al., 2008 [11]	NA	1. Active TB 2. LTBI	1. SF-36 2. DI	1. Physical component summary 2. Mental component summary 3. Depression	HRQoL decrements detected at the completion of therapy in TB patients
Unalan et al., 2008 [31]	NA	1. Active TB 2. Inactive tuberculosis	1. SF 36 2. BDI	1. Physical component summary (PCS) 2. Mental component summary 3. Social functionality	QoL decrements detected in active TB patients
Guo et al., 2008 (Canada) [21]	NA	1. Active TB 2. LTBI	1. SF-36 2. HUI2/3 3. VAS	Eight domain scores	For active TB and LTBI, health state utility values did not generate identical utility scores
Marra et al., 2004 (Canada) [58]	Multi-site study	Active TB patients	Focus groups and individual interviews	1. Physical functioning and 2. Emotional/mental well-being	QoL decrements detected in active TB patients
Dion et al., 2002 (Canada) [57]	NR	1. Active TB 2. Previous active TB patients	1. VAS 2. Standard gamble (SG)	1. Physical component summary (PCS) 2. Mental component summary (MCS)	QoL decrements detected in active TB patients

BDI: Beck Depression Inventory; BDQ: Brief Disability Questionnaire; EQ 5D: EuroQol; GHQ-12: 12-Item General Health Questionnaire; HUI: Health Utility Index; LTBI: Latent TB Infection; RHS-15: Refugee Health Screening-15; SF-36: Short Form-36; VAS: Visual Analog Scale; WHOQOL: World Health Organization Quality of Life.

**Table 2:** Characteristics of studies that assessed QoL in TB with HIV co-infection.

Study ID (Country)	Study Design	Participants	QoL Instrument Used	QoL Domain Assessed	Findings
Opollo et al., 2020 (Brazil, Haiti, India, Kenya, Malawi, Peru, South Africa, Uganda, Zambia, and Zimbabwe) [74]	Open-label trial	TB patient with advanced HIV infection	Questionnaire	1. Psycho- logical 2. Economic 3. Socio-cultural 4. Spiritual	HRQoL was found to improve over time, with no difference between arms
Hailu et al., 2020 (Ethiopia) [70]	Cross-sectional study	1. HIV mono-infected 2. TB and HIV co-infected patients	WHOQOL-HIV-BREF	1. Physical 2. Psychological 3. Level of independence 4. Social relationships 5. Environmental 6. Spiritual health	Poor HRQoL reported in TB/HIV co-infected patients in all domains compared with HIV mono-infected patients
Jha et al., 2019 (India) [71]	Cross-sectional study	1. HIV-TB co-infected patients 2. HIV patients	1. WHOQOL-HIV BREF 2. Beck’s Depression Inventory Scale	1. Physical health 2. Psychological well-being 3. Social relationship 4. Environmental health 5. Level of independence 6. Spiritual health	HIV-TB co-infected patients had a poorer QoL than only HIV patients
Mthiyane et al., 2016 (South Africa) [72]	Longitudinal study	HIV-TB co-infected patients	FAHI	1. Physical well-being 2. Functional 3. Global well-being 4. Emotional well-being 5. Cognitive functioning	Improvement in QoL consistent with a decrease in adverse events and signs and symptoms of TB
Deribew et al., 2013 (Ethiopia) [52]	Prospective study	1. HIV-infected patients without TB 2. TB/HIV co-infected patients	1. WHOQOL-HIV-BREF 2. Kessler-10 scale	1. Physical health 2. Psychological well-being 3. Social relationships 4. Environmental health 5. Level of independence 6. Spiritual health	Integrating mental health services into the TB/HIV programs can improve QoL
Dowdy et al., 2013 (Brazil) [69]	Cross-sectional study	1. HIV patients 2. TB patients 3. TB/HIV co-infected patients	1. Medical Outcomes Study HIV Health Survey (MOS-HIV) 2. Visual Analog Scale (VAS)	1. Physical health summary 2. Mental health summary 3. Visual Analog Scale	Among patients receiving treatment, those with HIV, active TB, and both conditions were found to have similar QoL
Louw et al., 2012 (Sout Africa) [54]	Cross-sectional study	1. TB patients 2. TB re-treatment patients 3. TB-HIV co-infected patients	Social Functioning (SF)-12 Health	1. Physical health Component 2. Mental health Component	TB and HIV weaken patient’s physical functioning and, thereby, impair QoL
Kittikraisak et al., 2012 (Thailand) [34]	Cross-sectional survey	1. TB patients 2. HIV-infected TB patients 3. HIV patients	1. EuroQol (EQ-5D) and 2. EuroQol Visual Analog Scale (EQ-VAS)	1. Mobility 2. Self-care 3. Usual activities 4. Pain or discomfort 5. Anxiety or depression	Patients with TB and HIV had impaired QoL
Neves et al., 2012 (Brazil) [73]	Cross-sectional study	1. HIV/TB co-infection patients 2. HIV-positive individuals without TB	WHOQOL-HIV-BREF	1. Physical 2. Psychological 3. Level of independence 4. Social relations	TB and HIV can alter biopsycho-social factors, which can impair QoL

**Table 3:** Characteristics of studies that assessed QoL in TB with comorbidities.

Study ID (Country)	Study Design	Participants	QoL Instrument Used	QoL Domain Assessed	Findings
Edwards et al., 2020 (Philippines) [76]	Cross-sectional survey	TB patients with comorbidity with undernutrition, diabetes (DM), and anemia.	WHOQOL-BREF questionnaire	1. Physical 2. Psychological 3. Social 4. Environmental	Food insecurity and nutritional status denote modifiable risk factors for poor QoL that may be improved through interventions
Yeung et al., 2016 [77]	Systematic reviews and meta-analyses	Populations with and without nontuberculous mycobacterial (NTM) disease	1. SF-362. SGRQ(St. George’s Respiratory Questionnaire 3. Visual analog scores (VAS)	1. Physical 2. Anxiety or depression 3. Pain or discomfort	Pulmonary NTM had worse health outcomes, thus impairing HRQoL

Nontuberculous mycobacterial (NTM).

## Data Availability

All relevant data supporting this study’s findings are within the manuscript.
